# DPED: Bio-inspired dual-pathway network for edge detection

**DOI:** 10.3389/fbioe.2022.1008140

**Published:** 2022-10-13

**Authors:** Yongliang Chen, Chuan Lin, Yakun Qiao

**Affiliations:** School of Automation, Guangxi University of Science and Technology, Liuzhou, China

**Keywords:** edge detection, vision pathway, swin transformer, convolutional neural network, deep learning

## Abstract

Edge detection is significant as the basis of high-level visual tasks. Most encoder-decoder edge detection methods used convolutional neural networks, such as VGG16 or Resnet, as the encoding network. Studies on designing decoding networks have achieved good results. Swin Transformer (Swin) has recently attracted much attention in various visual tasks as a possible alternative to convolutional neural networks. Physiological studies have shown that there are two visual pathways that converge in the visual cortex in the biological vision system, and that complex information transmission and communication is widespread. Inspired by the research on Swin and the biological vision pathway, we have designed a two-pathway encoding network. The first pathway network is the fine-tuned Swin; the second pathway network mainly comprises deep separable convolution. To simulate attention transmission and feature fusion between the first and second pathway networks, we have designed a second-pathway attention module and a pathways fusion module. Our proposed method outperforms the CNN-based SOTA method BDCN on BSDS500 datasets. Moreover, our proposed method and the Transformer-based SOTA method EDTER have their own performance advantages. In terms of FLOPs and FPS, our method has more benefits than EDTER.

## 1 Introduction

Edge detection is a low-level task in computer vision which is used in much computer vision processing (e.g., Image segmentation (Arbelaez et al., 2010; Mandal et al., 2014; Weng and Dong 2021), object recognition and detection (Ferrari et al., 2007; Girshick et al., 2014), optical flow (Demarcq et al., 2011; Chen and Wu 2015; Revaud et al., 2015; Liu et al., 2019), and sketch abstraction (Yu et al., 2017; Xie et al., 2019; Xu et al., 2022)]. Much excellent work has emerged in this field, from traditional edge detection methods (Canny 1986; Dollár and Zitnick 2014; Zhang et al., 2016) to the recently proposed deep CNN-based edge detection methods (Xie and Tu 2015; Liu et al., 2017; Wang et al., 2018; He et al., 2020). Edge detection is still a relatively open problem and new contributions are still to be made.

Transformer (Vaswani et al., 2017) has been introduced into the computational vision (CV) field due to its success in NLP and was soon widely used in CV in, for example, classification (Ramachandran et al., 2019; Dosovitskiy et al., 2020; Liu et al., 2021), detection (Carion et al., 2020; Liu et al., 2020; Touvron et al., 2021), and segmentation (Zheng et al., 2021). Recently, Pu et al. (Pu et al., 2022) used the Transformer architecture to build a new edge detection method called EDTER, which broke the dominance of CNN in the new computer vision field and became a new state-of-the-art edge detection method.

**FIGURE 1:**
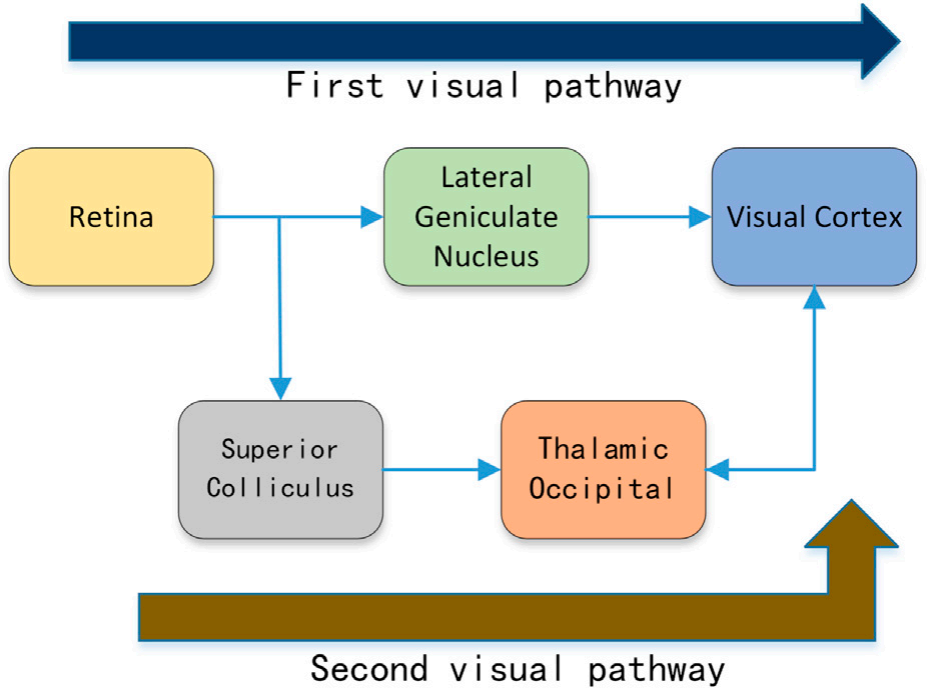
The dual-pathway structure from the retina to the visual cortex in the biological visual system.

The field of biology has also done much research on edge perception. Numerous studies (Hubel and Wiesel 1962; Hess et al., 2003; Loffler 2008) have shown that the visual cortex plays a crucial role in edge detection and processing. Many studies have proposed bio-inspired edge detection algorithms (Li 1998; Yen and Finkel 1998; Grigorescu et al., 2003; La Cara and Ursino 2008; Yang et al., 2014; Tang et al., 2016) by simulating the neural cell response pattern in the visual cortex. The dual-pathway structure of the biological visual system promotes information processing and information exchange in the visual cortex. The information from the retina is divided into two flows, which undergo different processing and finally merge in the visual cortex. As shown in Figure 1, part of the information from the retina is processed by the lateral geniculate nucleus (LGN) and transferred to the visual cortex; other information from the retina is processed by the superior colliculus cells and then passed through the thalamus-occipital transmutation. These two pathways are called the first visual pathway and the second visual pathway, respectively.

Most of the information entering the visual cortex comes from the first visual pathway, which forms the visual backbone. However, the residual visual phenomenon (Pöppel et al., 1973) shows that the second visual pathway can also transmit information to the visual cortex, thereby triggering visual perception. Luck et al. (Luck et al., 1997) found that attention exists in the visual cortex. Yan et al. (Yan et al., 2018) found that attention in the visual cortex can be divided into early attention and late attention according to the occurrence time. White et al. (White et al., 2017a; White et al., 2017b) have shown that cells in the superior colliculus in the second visual pathway can generate attentional information. It is earlier than attention in the visual cortex and may be transmitted to the visual cortex through connections.

Inspired by these studies, we propose a dual-pathway edge detection method (DPED), consisting of a two-pathway encoding network and a decoding network (DN), like the biological vision system. The two-pathway encoding network consists of a first-pathway network (FPN) and a second-pathway network (SPN). As the information backbone network, FPN undertakes the function of extracting multi-scale features and is deeper and more complex. SPN is a shallow network with a simple structure. To simulate the attention transmission and feature fusion between two visual pathways, we designed a second-pathway attention module (SPAM) and pathways fusion module (FPM), which are placed between the FPN and SPN. To make full use of the features extracted by the encoding network, we designed a feature fusion module (FFM) as the basic module for constructing the decoding network.

Our contributions can be summarized as follows. 1) We propose an edge detection method, the dual-pathway edge detection method (DPED, based on Swin Transformer and depth-wise separable convolution. 2) Inspired by biological visual pathways, we designed a second-pathway network (SPN). We designed an SPAM and a pathways fusion module (PFM) to simulate the information exchange between the first and the second visual pathways. Experiments show that SPN combined with SPAM and PFM can also improve the performance of other edge detection methods. 3) Without adding an additional dataset for training, our method can achieve better results on the BSDS500 dataset. We have made a reasonable explanation for this phenomenon.

Extensive experiments indicate that our proposed method outperforms previous CNN-based edge detection methods on three well-known datasets. Compared with the state-of-the-art method EDTER, our method—while some performance is slightly lower—has a lower computational cost and faster inference speed.

## 2 Related work

For original research articles, please note that the Material and Methods section can be placed in any of the following ways: before Results, before Discussion, or after Discussion.

### 2.1 Bio-inspired edge detection

Numerous studies (Hubel and Wiesel 1962; Hess et al., 2003; Loffler 2008) have shown that the visual cortex plays a crucial role in edge detection and processing. Many bio-inspired edge detection methods started from studies on the visual cortex. Hubel and Wiesel et al. (Hubel and Wiesel 1962) show that V1 visual cortex neurons are sensitive to lines and edges in the classical receptive field (CRF) and that areas beyond CRF produce an inhibitory effect—nonclassical receptive field (non-CRF) inhibition. Grigorescu et al. (Grigorescu et al., 2003) proposed an anisotropic model and an isotropic model. They used the two-dimensional Gabor function to model the CRF of a central cell, while using the difference of Gaussian functions to model the non-CRF inhibitory. Compared to traditional detection methods, this model dramatically improves the accuracy of contour detection but suppresses the contours of local regions. Tang et al. (Tang et al., 2007) proposed a butterfly suppression model, which divides a circular suppression area into two suppressor sub-regions and two facilitator sub-regions. This model selectively preserves the edge part while suppressing its texture. Lin et al. (Lin et al., 2018) proposed a nonlinear inhibition model that partitions multiple subunits within a circular inhibition region. In terms of large-scale computing, the model effectively reduces the computational cost and somewhat improves performance. Yang et al. (Yang et al. (2013) proposed a color-opponent (CO) model based on color cues which uses an opponent mechanism to detect brightness edges. Yang et al. (Yang et al., 2015) then proposed the SCO model to suppress the texture information, which merged the spatial sparsity constraint with the CO model. Akbarinia et al. (Akbarinia et al., 2017) proposed the ASM model in which the relationship between the center and the surround of the receptive field is adjusted according to the intensity of the stimulus. Tang et al. (Tang et al., 2019) proposed an edge detection method that combines bio-inspired methods and deep learning frameworks and provides a new approach to integrating brain cognition research into neural networks.

### 2.2 CNN-based edge detection

With the development of deep learning, the end-to-end edge detection method has gradually replaced traditional hand-designed edge detection methods. Xie et al. (Xie and Tu 2015) proposed holistically-nested edge detection (HED), which can achieve end-to-end training and prediction through VGG16. After convolution processing, all the side outputs are interpolated into the same size to predict the final edge map. Wang et al. (Wang et al., 2018) then found that the edge image of HED is too thick and hope to solve the problem of the rough edge of HED network output by designing an exemplary decoding module for the top-down fusion of features. Liu et al. (Liu et al., 2017) argued that edge detection needs richer feature information, which fuses the multi-layer feature information of the VGG16 network and proposed RCF. He et al. (He et al., 2020) found that the edge thickness of different locations in the dataset differs, and proposed the BDCN, which allows each stage to predict different scales of edge information through a two-way connection. Cao et al. (Cao et al., 2020) proposed the deep refinement network (DRC) by designing a refinement module to build a decoding network to achieve the fusion of features with different scales. Lin et al. (Lin et al., 2020) proposed a new edge detection method (lateral reinforcement network for contour detection—LRC). They used the decoding module to design a deeper decoding network to fuse multi-scale features. Deng et al. (Deng and Liu 2020) proposed deep structure contour detection (DSCD). Differing from previous work, they proposed adding a new module between the encoding network and the decoding network and proposed a new loss function, achieving good results.

### 2.3 Vision transformer

Recently, as an alternative to CNN, Transformer (Vaswani et al., 2017) has been introduced to the field of computer vision for image classification (Ramachandran et al., 2019; Kolesnikov et al., 2021; Liu et al., 2021), object detection (Carion et al., 2020; Touvron et al., 2021), and segmentation (Zheng et al., 2021). Transformer architecture has been used to explore the relationship between different regions of an image in learning to focus on important regions. Vision Transformers handle the input image by usually cutting it into fixed-size patches (e.g., 16*16). This method is acceptable for the coarse-grained task of image classification; however, it is not very suitable for fine-grained tasks such as edge detection and semantic segmentation at the pixel level. Liu et al. (Liu et al., 2021) established Swin Transformer by introducing the shift window attention, thus solving the high complexity of self-attention computing. The excellent performance of Transformer quickly attracted the attention of researchers in the field of edge detection, and Transformer-based methods began to appear. Recently, Pu et al. (Pu et al., 2022) proposed edge detection with Transformer (EDTER), which extracts features in two stages. The first stage captures the long-range global context on coarse-grained image patches. Then, in the second stage, short-range local cues are mined in fine-grained patches.

In summary, we find that the current Transformer, as a possible alternative to CNN, is gradually proving its performance in various vision tasks. In this study, we try to build a new edge detection method based on Swin Transformer. Inspired by the research on biological visual pathways and previous CNN-based edge detection methods, we designed a dual-pathway edge detection method (DPED).

## 3 Methods

In this section, we describe the specific details of our proposed method. We firstly introduce the research on biological visual pathways, and then introduce the architecture of our network.

### 3.1 Biological vision system

According to the neuroanatomy of the visual system, the flow of information in the human visual system is not a straight line. The information encoded from the retina goes through different processing which finally flows into the visual cortex. As shown in Figure 1, part of the information encoded by the retina is transmitted to the visual cortex through the lateral geniculate nucleus, which is called the first visual pathway. The other part of the information from the retina is processed by the superior colliculus cells and then transferred to the visual cortex through the thalamic occipital—this pathway is called the second visual pathway.

In neuroscience, it is generally believed that the first visual pathway is the backbone of visual information. However, the phenomenon of residual visual function (Pöppel et al., 1973) shows that the second visual pathway also transmits information that can produce visual perception to the visual cortex. Luck et al. (Luck et al., 1997) found selective attention in the visual cortex. Yan et al. (Yan et al., 2018) found that the attention in the visual cortex can be divided into the early attention components and the late attention components according to the occurrence time. White et al. (White et al., 2017a; White et al., 2017b) showed that the superficial cells of the superior colliculus in the second visual pathway can generate meaningful information to measure the importance of external information. The information generated by the second visual pathway is earlier than that in the visual cortex. The visual information produced by the second visual pathway may be projected to the other visual cortex through rich connections.

Inspired by these biological vision studies, the second-pathway network (SPN) is designed outside the backbone network (first-pathway network—FPN). On the one hand, SPN can provide early attention to the backbone network (FPN) and the early attention can work with late attention in FPN to improve coding efficiency; on the other hand, the features extracted by SPN and FPN are fused to obtain the dual-pathway feature.

### 3.2 Network architecture

**FIGURE 2:**
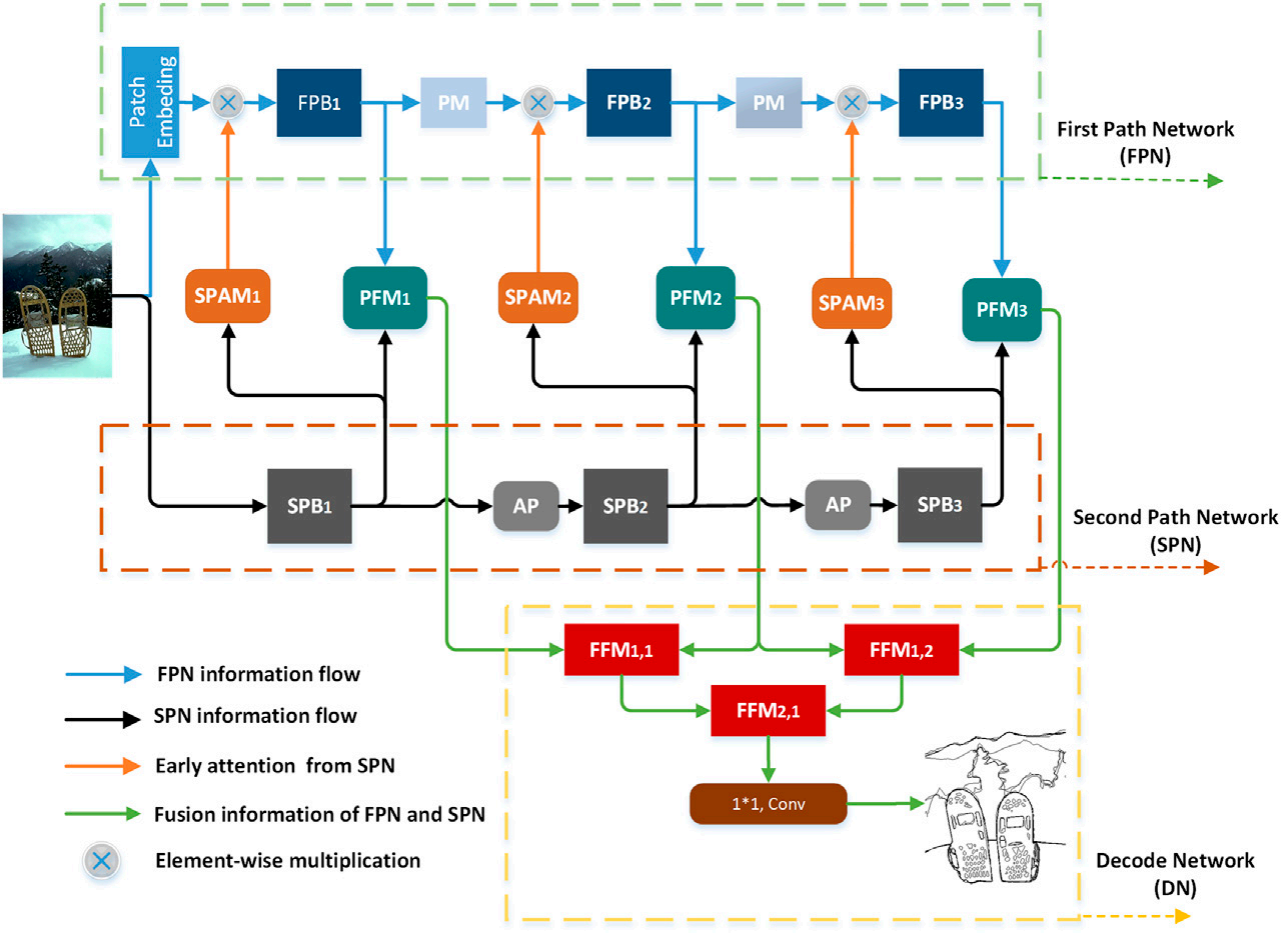
The overall structure of the network.

According to Figure 2, the DPED network consists of three parts: the FPN (green dotted frame), the SPN (brown dashed frame) and the decoding network (DN—orange dotted frame). We can briefly introduce the flow of feature information in the network. When the image enters the network, it will be encoded by FPN and SPN into two feature flows: the feature flow of FPN (blue arrow) and that of SPN (black arrow). The feature flow of SPN through the SPAM can generate early attention information (orange arrow) to guide FPN to extract features. Then, the feature flow of FPN and the feature flow of SPN are fused to achieve the dual-pathway feature flow (green arrow) through the pathways fusion module (PFM) and is sent to DN for decoding. Finally, it is compressed by a 1 × 1 conv to generate the edge probability map.

#### 3.2.1 The first-pathway network (FPN)

**FIGURE 3:**
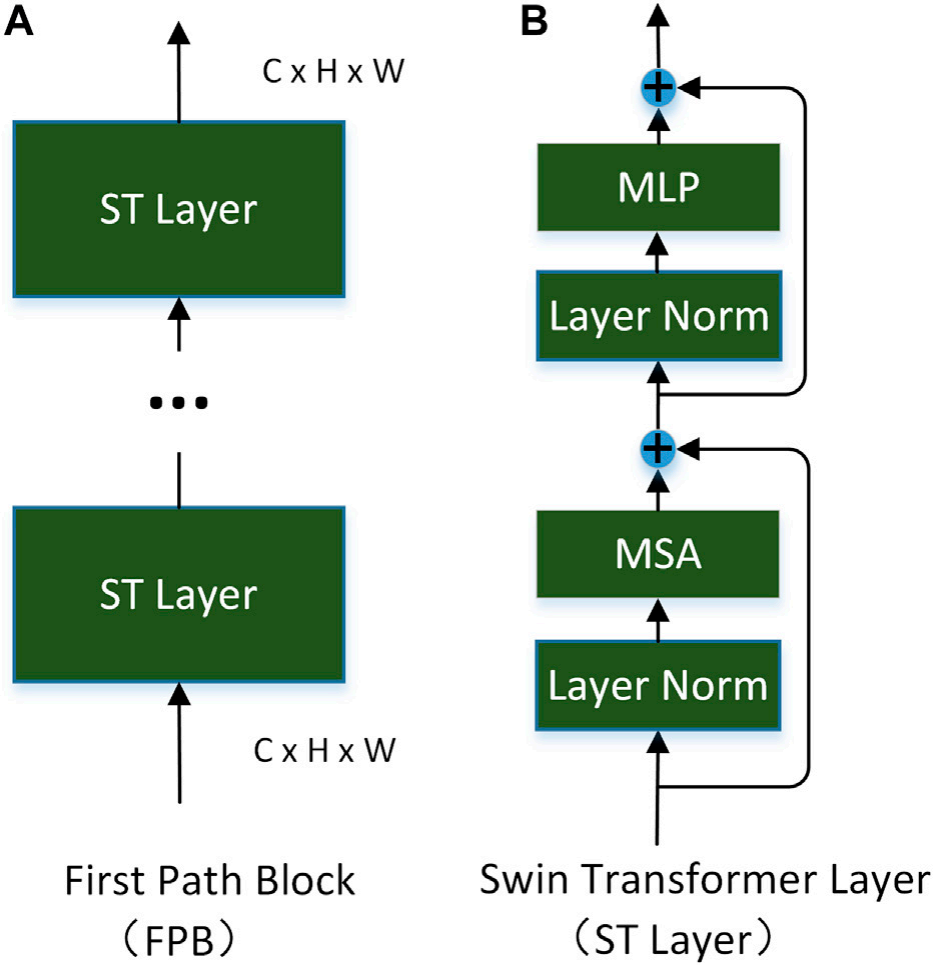
Modules used in the first-pathway network.

**FPN** consists of three first-pathway blocks (FPB). The specific structure of a FPB is shown in Figure 3A. Each is composed of a Swin Transformer layer (ST layer) (Liu et al., 2021). The number of ST layers in three FPBs is 2, 2 and 18 respectively. The structure of the ST layer is shown in Figure 3B, which is described later.

Research (Hubel and Wiesel 1962; Hess et al., 2003; Loffler 2008) has shown that the lateral connection of primary cortex neurons in the first visual pathway plays a crucial role in edge detection and that selective attention mechanism exists in V1. Using these insights, we use the ST layer as the foundation of our first-pathway block. The specific structure of the ST layer is shown in Figure 3B. In contrast to convolution, Lin (Lin et al., 2021) demonstrated that, in Transformer, the convolution kernel dynamically changes according to the input. It can simulate the lateral connections of the visual cortex. Inspired by the fact that the occurrence of attention in the second visual pathway is earlier than that in the visual cortex, FPN will be guided by the early attention (EA) generated from SPN. Given the input image $I\in R^{3\times H\times W}$ (H, W represents the height and width of the image, respectively, and 3 represents the number of channels of the image), the first-pathway feature $F_{1}$ is calculated as follows:

$$\begin{array}{c} F_{1,0}=PE\left(I\right),\;\\ {\;F}_{1,i}={PM(FPB}_{i}\;({{EA}_{i}*\;F}_{2,i-1})) \end{array}$$ (1)

where, $F_{1,0}\in R^{\left(4*C\right)\times \frac{H}{4}\times \frac{W}{4}},{\;F}_{1,i}\in R^{\left(4*i*C\right)\times \frac{H}{2^{i+1}}\times \frac{W}{2^{i+1}}}\;,\;i=1,2,3.$ This represents the features extracted by the i -th stage of FPN. The specific calculation of EA is shown in Eq. 6. PE is a patch embedding operation, which projects each bit of patch information of a picture into a high-dimensional space to transform token information. PM is a patch merging operation, which can down-sample the feature information consistent with PE and PM in Swin (Liu et al., 2021).

**The Swin Transformer Layer (ST layer)** (Liu et al., 2021) solves the high complexity of the original standard in the Transformer layer (Vaswani et al., 2017) by introducing the self-attention of local windows and the mechanism of moving windows. The concrete structure of the ST layer is shown in Figure 3B. At the same time, long-distance relationships can still be modeled by hierarchical stacking. Given the input image $I\in R^{3\times H\times W}$ , the specific operation of Swin Transformer is to divide the input into several non-overlapping K×K local windows. For the feature X in each K×K window, the query, key, and value are calculated as follows:

$$\begin{array}{c} Q=W_{q}X,\;K=W_{k}X,\;V=W_{v}X \end{array}$$ (2)

where $W_{q},W_{k},W_{v}$ represent different mapping matrices. The mapped features Q, K and V are used to calculate the self-attention matrix as follows:

$$\begin{array}{c} Attention\left(Q,K,V\right)=softmax\left(\frac{QK^{T}}{\sqrt{d\;}}+B\;\right)V \end{array}$$ (3)

where B is a relative position bias that can be learned; the original Transformer layer will calculate the self-attention several times in parallel—called multi-head self-attention (MSA). The feature information of each window is better extracted by MSA and a multi-layer perceptron (MLP) with a GELU activation function. Before entering MSA and MLP, layer norm (LN) and residuals connect are required. The specific operation is as follows:

$$\begin{array}{c} X=MSA\left(\mathrm{LN}\left(X\right)\right)+X,\;\\ X=MLP\left(\mathrm{LN}\left(X\right)\right)+X \end{array}$$ (4)

In Swin, the MSA is changed to window multi-head self-attention (W-MSA) and shift window multi-head self-attention (SW-MSA). There is no cross-window connection in a ST layer. To establish the long-term relationship of feature information, W-MSA and SW-MSA are used interchangeably in constructing the network—see the original study (Liu et al., 2021) for details.

#### 3.2.2 The second-pathway network (SPN)

**FIGURE 4:**
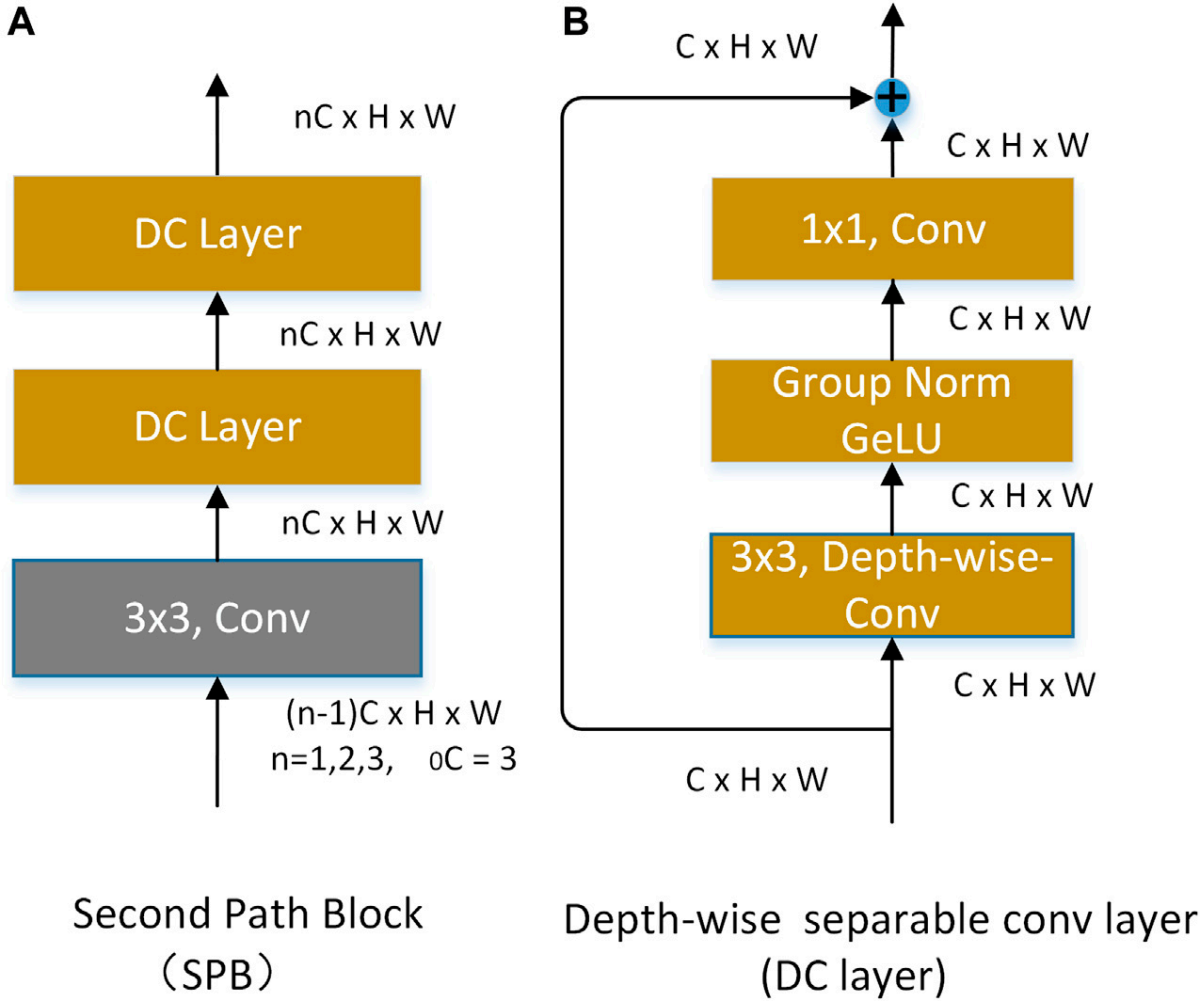
Modules used in the second-pathway network.

**The SPN** consists of three the second-pathway blocks (SPB). The structure of a SPB is shown in Figure 4A. Each SPB consists of a basic 3 × 3 ordinary convolution and two 3 × 3 depth-wise separable convolutions (Chollet 2017) with residual structure. The structure of the DC layer is shown in Figure 4B. Considering the center-surround receptive field of the superficial cells, we take the residual shortcut and 3 × 3 depth-wise separable convolution as the central feature and the peripheral feature, respectively. At the same time, 3 × 3 depth-wise separable convolution has fewer parameters.

When an input image $I\in R^{3\times H\times W}$ is given, the multi-scale features can be obtained after each stage of SPB in SPN and the second-pathway feature $F_{2}$ is calculated explicitly as follows:

$$\begin{array}{c} F_{2,1}={SPB}_{1}\left(\;I\;\right),\;\\ {\;F}_{2,i}={SPB}_{i}\left(AP\left(F_{2,i-1}\right)\right) \end{array}$$ (5)

where $F_{2,i}\in R^{\left(i*C\right)\times \frac{H}{2^{\left(i-1\right)}}\times \frac{W}{2^{\left(i-1\right)}}},\;i=1,\;2,3.$ This represents the features extracted by the i -th SPB in SPN. AP represents average pooling.

#### 3.2.3 Feature transmission and fusion between FPN and SPN

Attention can be generated in the second visual pathway of the biological visual system; its occurrence time is earlier than in the visual cortex. Inspired by these studies, we transform the features $F_{2,i}$ extracted from SPN into early attention (EA) to FPN through the SPAM. Inspired by the phenomenon of residual visual function, we fuse the feature $F_{1,i},\;{\;F}_{2,i}\;,\;i=1,2,3.$ extracted by FPN and SPN through the pathways fusion module PFM to get the dual-pathway feature $D_{i}$ .

**FIGURE 5:**
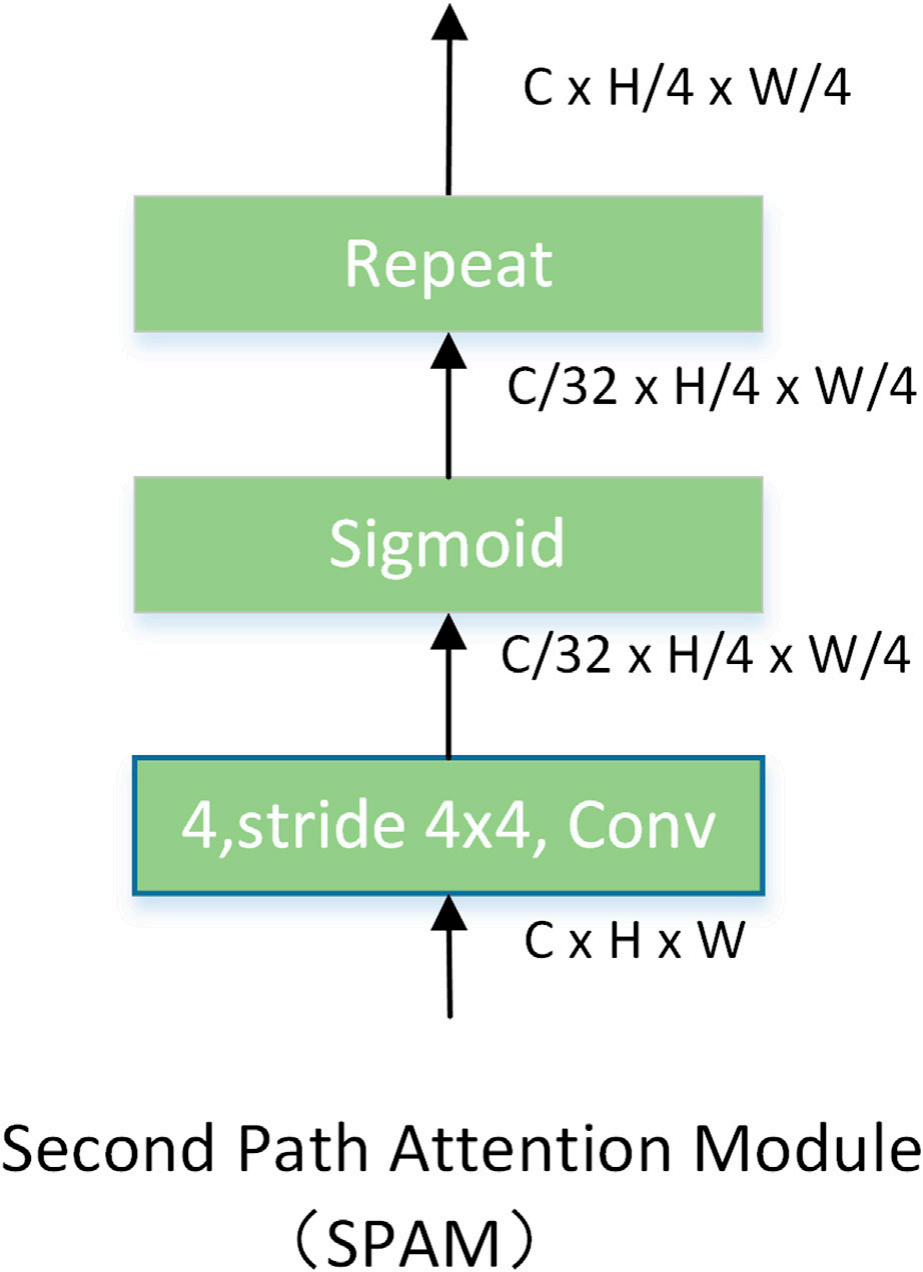
Modules used by the second-pathway network to generate attention.

**The second-pathway attention module.** The structure of SPAM is shown in Figure 5, consisting of a convolution with a stride of four convolution kernels of 4 × 4 and a sigmoid activation function. The features of every 32 channels in FPN share the same attention. Hence, we need to repeat the attention to keep the dimension of ${EA}_{i}$ and $F_{1,i}$ consistent. The attention map EA is calculated as follows:

$$\begin{array}{c} {EA}_{i}={SPAM}_{i}\left(F_{2,i}\right) \end{array}$$ (6)

where ${EA}_{i}\in R^{\left(4*i*C\right)\times \frac{H}{2^{i+1}}\times \frac{W}{2^{i+1}}},\;i=1,2,3$ , representing the early attention generated by i -th stage of SPN.

**FIGURE 6:**
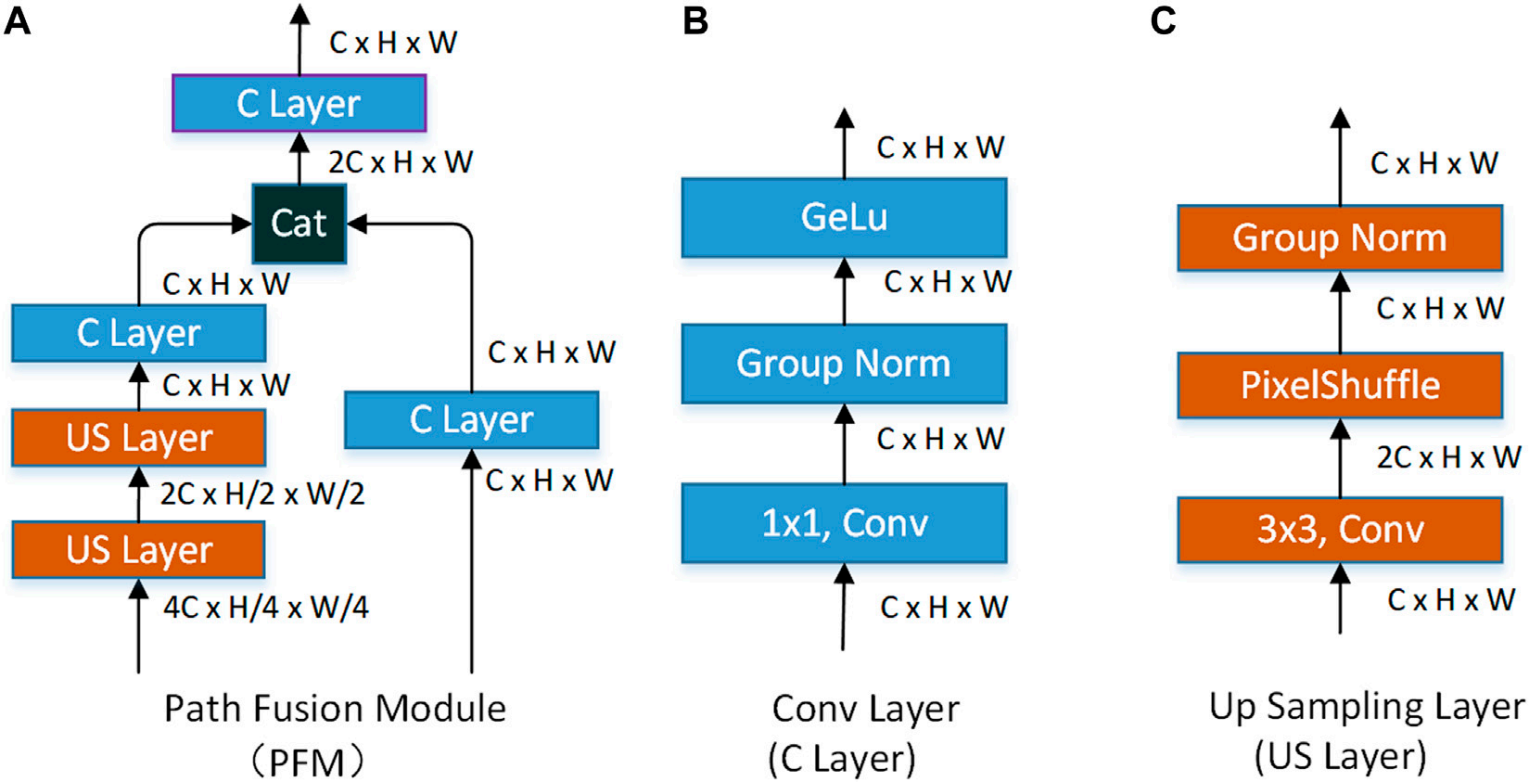
Modules used to fuse features extracted by FPN and features extracted by SPN.

**The pathways fusion module (PFM).** The function of PFM is to fuse the feature information extracted by FPN and SPN. The specific structure of PFM is shown in Figure 6A, which consists of a conv layer (C layer) and up-sampling layer (US layer). The specific structure of the C layer is shown in Figure 6B, which consists of a 1 × 1 convolution, group normal and GELU activation functions. The structure of the US layer is shown in Figure 6C. Compared with the current popular methods (Liu et al., 2017; Cao et al., 2020; He et al., 2020; Lin et al., 2020), we do not use the deconvolution for up-sampling but choose the sub-pixel convolution method to build the US layer. Considering the down-sampling of patch merging, which increases the number of channels and decreases the resolution, we use the sub-pixel convolution for up-sampling. Group normalization is adopted after the pixel shuffle.

PFM accepts the features extracted by FPN and SPN. With the help of PFM, we fuse the multi-scale feature extracted by FPN and SPN ( $F_{1,i},\;{\;F}_{2,i}\;,\;i=1,2,3$ ),

$$\begin{array}{c} D_{i}={PFM}_{i}\left(F_{1,i},\;{\;F}_{2,i}\right) \end{array}$$ (7)

where $D_{i}\in R^{\left(i*C\right)\times \frac{H}{2^{\left(i-1\right)}}\times \frac{W}{2^{\left(i-1\right)}}},\;i=1,2,3$ is dual-pathway feature $D_{i}$ , which is the fusion of the features extracted by FPN and SPN.

#### 3.2.4 Decoding network

Recently, an excellent edge detection method (Wang et al., 2018; Cao et al., 2020; Lin et al., 2020) obtained richer feature information by fusing the multi-scale features extracted by the backbone. LRC (Lin et al., 2020) shows that hierarchical fusion through a fusion module is beneficial for achieving more abundant features. In this study, the design of a decoding network refers to the hierarchical fusion method.

**FIGURE 7:**
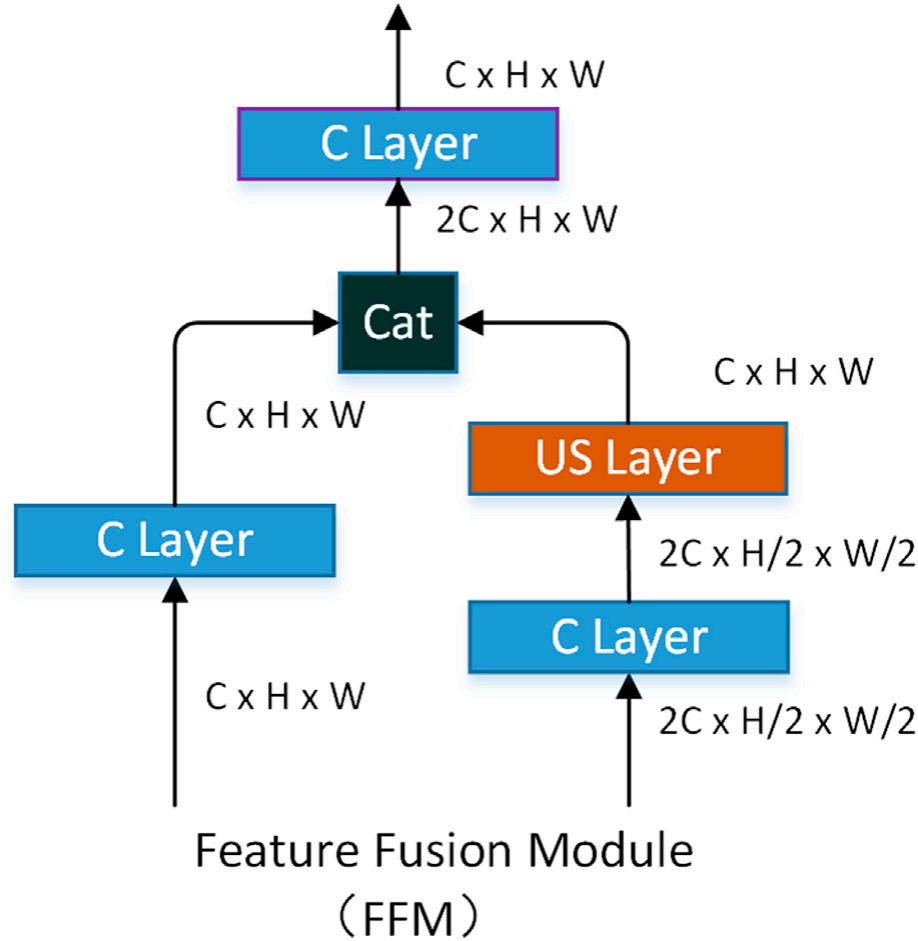
Decode modules used in the network.

The decoding network of our method consists of the feature fusion module (FFM). The role of the FFM is to fuse features of different sizes together to obtain richer edge features. FFM accepts both high-channel low-resolution feature and low-channel high-resolution feature. The low-channel high-resolution feature is processed by the C layer mentioned earlier. The low-channel high-resolution feature is first processed by the C layer and then unsampled by the US layer to obtain a high-resolution feature. Finally, two features with the same resolution are connected according to the direction of the channel, and the number of channels of the feature is then compressed by a C layer. The specific structure of FFM is shown in Figure 7. ${FFM}_{l,i}$ fuses the two adjacent features to obtain the next level of features, and l is iteratively calculated as follows:

$$\begin{array}{c} D_{l,i}={FFM}_{l,i}\left(D_{l-1,i},\;D_{l-1,i+1}\right),\;\\ l=1,\;2,\;i=1\ldots \left(3-l\right) \end{array}$$ (8)

where $D_{l,i}\in R^{\left(i*C\right)\times \frac{H}{2^{\left(i-1\right)}}\times \frac{W}{2^{\left(i-1\right)}}},\;D_{0,\;i}=D_{i}.$ l represents l -th level fusion, and i represents the i -th feature of l -th level fusion. The fusion feature of the last level $D_{2,\;1}$ passes through a 1 × 1 convolution and sigmoid to obtain the probability map of the final output, which is formulated as,

$$\begin{array}{c} E=Sigmoid\left(Conv\left(D_{2,\;1}\right)\right) \end{array}$$ (9)

where $E\in R^{1\times H\times W}$ represents the final output of DPED.

## 4 Experiments

In this section, we will verify the effectiveness of our proposed method through ablation experiments. At the same time, we explore the generalization of our proposed method in other methods. Finally, our method is compared with other methods on different public datasets.

For comparison, we use three evaluation metrics to measure the performance of edge detection methods. The evaluation metrics include the F-score with the thresholds of optimal dataset scale (ODS), F-score with the thresholds of optimal image scale (OIS), and average precision (AP). According to previous work (Xie and Tu 2015; Liu et al., 2017; He et al., 2020), the maximum allowable distance between the positioning tolerance control edge result and ground truth is set to 0.0075 for BSDS500 and Multicue and to 0.011 for NYUDv2.

### 4.1 Experimental dataset

We evaluated our method on three public datasets in the field of edge detection, including BSDS500 (Arbelaez et al., 2010), NYUD-v2 (Silberman et al., 2012), and Multicue (Mély et al., 2016). BSDS500 is the most classic dataset in edge detection, containing 200 training images, 100 validation images, and 200 testing images. Each image is annotated by multiple people. Following previous work (Xie and Tu 2015; Deng et al., 2018; Wang et al., 2018; Tang et al., 2019; Cao et al., 2020; He et al., 2020), we used the same method for the augmentation training set and validation set as the training set. We added an extra PASCAL VOC Context dataset to BSDS500 as a training dataset in some experiments. NYUDv2 contains 1,449 depth aligned RGB images, which are split into 381 training, 414 validation, and 654 testing images. To facilitate comparative experiments, we also augmented the dataset with the data methods used previously (Xie and Tu 2015; Liu et al., 2017; Lin et al., 2020). Multicue contains 100 natural scenes, each with a sequence of two frames from different angles. The last frame in the left view is marked with edges and boundaries. Consistent with previous work (Xie and Tu 2015; Liu et al., 2017; He et al., 2020), we randomly divided 100 annotation frames into 80 and 20 frames for the training and testing.

### 4.2 Implementation details

**Life Science Identifiers.** We implemented our network using Pytorch, a well-known platform in the community. The first-pathway network (FPN) is initialized by a partial weight of the Swin Transformer model pre-trained in the ImageNet dataset. The other part is initialized randomly. We changed the window size of W-MSA and SW-MSA from 7 to 8 because we wanted to keep the receptive field larger when there are only three stages in the network. To enable the network to process images of any size, the resolution of the input image was padded to a size divisible by 128 and these padding elements were removed from the network output.

The loss function is consistent with recent work. The threshold γ and weight coefficient λ in the loss function are set to 0.17 and 5 on the multi-person labeled dataset BSDS500, respectively. There is no need to set the threshold for the binary annotated dataset NYUDv2. Because the scaling and rotation in the data augmentation will bring additional edge annotations, we used threshold and weight coefficients consistent with those in BSDS500. Due to the Multicue dataset including high-resolution images, we randomly cropped 500 × 500 patches from each image. We set γ and λ as 0.17 and 5, respectively.

The hyperparameter of all experiments is set as follows: batch size (1), learning rate (1e−6) momentum (0.9), snf weight decay (2e−4). We used the SGD optimizer to train four epochs on the BSDS500 dataset. The learning rate is adjusted from the second epoch and is then divided by 10 at each completed epoch. For the training on NYUD-v2 data sets, we used an SGD optimizer for 45 epochs. The learning rate decreases from 20 epochs and then is divided by 10 at each completed epoch. We train 200 epochs on the Multicue dataset. The learning rate is adjusted from the 100 epoch and is then divided by 10 at each completed epoch.

To ensure the accuracy and reproducibility of the experiment, the random seed in all experiments is fixed at 78.

### 4.3 Ablation experiment

In this section, we firstly used the BSDS500 training and validation set for training and evaluated our method on the test set. The first group of experiments explored the validity of SPAM and PFM. The second group of experiments verified the generalization of SPN in other methods. The third group of experiments verified the effectiveness of our designed FFM. The fourth group of experiments explored the impact of adding additional PASCAL VOC Context data sets (VOC) to the BSDS500 dataset for training in our method.

Experiment 1: We explored the validity of SPAM and PFM. SPN works with SPAM and PFM. If both are removed, SPN is removed. The experimental results are summarized in Table 1.We found that SPAM can slightly improve ODS and AP, and OIS is flat or down. PFM can significantly improve ODS and OIS, but AP is down. We believe that the addition of PFM can make the feature extracted from the SPN participate in the generation of the final edge map. Due to the simple structure and small parameters of SPN, the extracted features not only contain meaningful and detailed edge information but also much textural information, which leads to a significant decrease in AP while ODS and OIS are improved. Using SPAM and PFM together, the single-scale ODS performance is improved by 0.007 and the multi-scale ODS performance is improved by 0.006. The OIS and the AP metrics are improved to varying degrees.

**TABLE 1:** Validity of SPAM and PFM in DPED. W/o-SPAM means the removal of the SPAM module from the DPED. W/o-PFM means the removal of the PFM module from DPED. SS means single-scale testing and MS means for multi-scale testing. In bold are the best results of experiments.

Method		ODS	OIS	AP
DPED-w/o-SPAM-w/o-PFM	SS	0.827	0.845	0.880
DPED-w/o-SPAM-w/o-PFM	MS	0.840	0.861	0.831
DPED-w/o-PFM	SS	0.829	0.845	0.883
DPED-w/o-PFM	MS	0.842	0.858	**0.857**
DPED-w/o-SPAM	SS	0.832	0.848	0.877
DPED-w/o-SPAM	MS	0.845	0.861	0.792
DPED	SS	**0.834**	**0.850**	**0.884**
DPED	MS	**0.846**	**0.861**	0.838

Experiment 2: To verify the generalization of SPN, we selected HED (Xie and Tu 2015), LPCB(Deng, Shen, Liu, Wang and Liu 2018), and LRC (Lin, Cui, Li and Cao 2020) as experimental networks and connected SPN to these for experimentation through SPAM and PFM. The experimental summary is shown in Table 2. The experiments show that our proposed SPN can improve the ODS, OIS, and AP of LPCB and LRC networks to varying degrees. However, SPN can improve the ODS and OIS of HED, but AP decreases a lot. We think that the decoder of HED is too simple. The fusion feature of SPN cannot be well-processed. In the LRC network with a more complex decoding network, the SPN has greatly improved the three evaluation indicators.

**TABLE 2:** Generalization of SPN in other networks. SS stands for single-scale. MS stands for multi-scale. √ indicates that SPN is connected to the backbone through SPAM and PFM. × indicates that no changes are made. In bold are the best results of experiments.

Method	SPN	ODS	OIS	AP
HED-SS Xie and Tu. (2015)	×	0.782	0.804	**0.833**
HED-SS Xie and Tu. (2015)	√	**0.791**	**0.809**	0.813
LPCB-SS Deng et al. (2018)	×	0.800	0.816	-
LPCB-SS Deng et al. (2018)	√	**0.803**	**0.823**	0.856
LPCB-MS Deng et al. (2018)	×	0.808	0.824	-
LPCB-MS Deng et al. (2018)	√	**0.815**	**0.836**	0.867
LRC-SS Lin et al. (2020)	×	0.792	0.813	0.808
LRC-SS Lin et al. (2020)	√	**0.803**	**0.824**	**0.836**
LRC-MS Lin et al. (2020)	×	0.808	0.830	0.849
LRC-MS Lin et al. (2020)	√	**0.815**	**0.835**	**0.863**

Experiment 3: To explore the validity of the feature fusion module (FFM), we used the refinement block proposed in LRC (Lin et al., 2020) to replace our FFM. The experimental results are summarized in Table 3. The experiments show that our FFM has a better effect than the refine block proposed in LRC.

**TABLE 3:** Validity of FFM in DPED. SS stands for single-scale. MS stands for multi-scale, × represents the replacement of the FFM with a refine block, √ represents the use of FFM. In bold are the best results of experiments.

Method	FFM	ODS	OIS	AP
DPED-SS	×	0.829	0.845	0.861
DPED-SS	√	**0.834**	**0.850**	**0.884**
DPED-MS	×	0.841	0.857	0.863
DPED-MS	√	**0.846**	**0.861**	0.838

Experiment 4: We explored the impact of adding the VOC dataset to BSDS500 for experiments in our method. The experimental results are summarized in Table 4. According to the experiments, our method can achieve better results with fewer data. The results of our method trained only on the BSDS500 dataset are better than those trained on the BSDS500 with the VOC dataset.

**TABLE 4:** The impact of adding the VOC dataset to the BSDS500 dataset on our proposed method. SS stands for single-scale. MS stands for multi-scale. √ indicates that the PASCAL VOC context dataset is added to the training set. × indicates that no changes are made. In bold are the best results of experiments.

Method	VOC	ODS	OIS	AP
DPED-SS	×	**0.834**	**0.850**	**0.884**
DPED-SS	√	0.823	0.840	0.832
DPED-MS	×	**0.846**	**0.861**	**0.838**
DPED-MS	√	0.843	0.860	0.775

**FIGURE 8:**
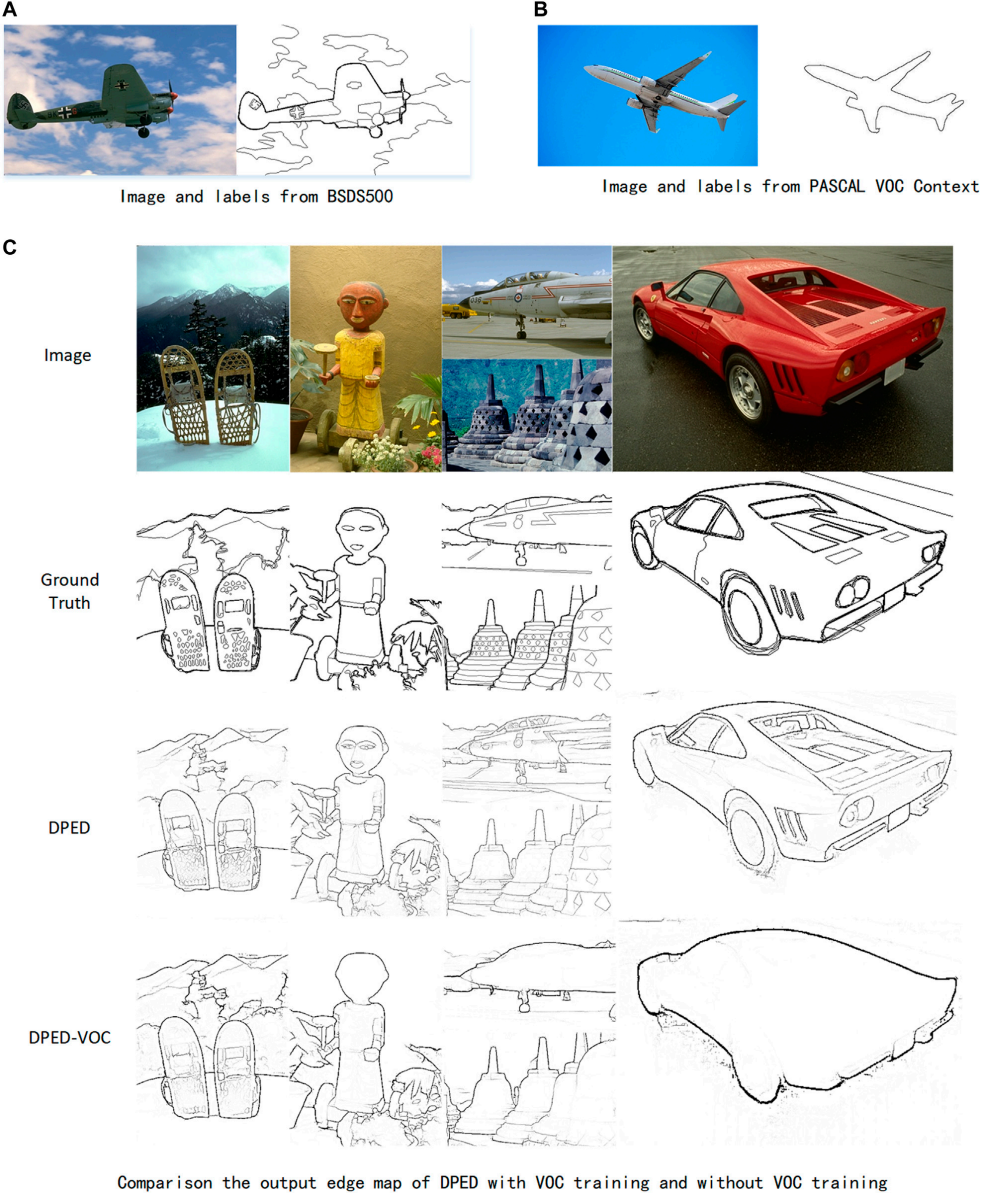
Comparison of the output edge map of DPED with VOC training and without VOC training. **(A)** are pictures and edge labels in BSDS500, and **(B)** are pictures and edge labels in the VOC dataset. It can be clearly seen that the two labels are very different and even ambiguous. In **(C)**, VOC represents adding additional VOC datasets for training. It can be found that adding the VOC dataset for training will cause network output far from ground truth.

The previous method (Liu et al., 2017; He et al., 2020) assumed that adding the VOC dataset to the BSDS500 dataset for training can suppress meaningless textural information and output a cleaner edge map to improve network performance. Figures 8A,B shows the difference and ambiguity between the annotation method of the VOC dataset and the annotation method of BSDS500. The BSDS500 annotation has some detailed information but the VOC dataset annotation ignores all the detail. Adding these ambiguously labeled data will mislead our method and the output results of the proposed method also confirm our viewpoint. As shown in Figure 8C, the output edge map of our method is far from the ground truth of the BSDS500 dataset (full of semantic detail annotations) and close to the ground truth of the VOC dataset (lack of detail annotations).

### 4.4 Comparison with other models

**BSDS500 Dataset.** We conducted testing on BSDS500 and compared it with some traditional edge detection methods and deep learning edge detection methods, including Canny (Canny 1986), SCO (Yang et al., 2015), SED (Akbarinia and Parraga 2018), gPb(Arbelaez et al., 2010), OEF (Hallman and Fowlkes 2015), SE (Dollár and Zitnick 2014), COB (Maninis et al., 2016), HED (Xie and Tu 2015), RCF (Liu et al., 2017), CED (Wang et al., 2018), LRC (Lin et al., 2020), BDCN (He et al., 2020), and EDTER (Pu et al., 2022). We mixed training and validation sets as training data and evaluated on the testing set.

**FIGURE 9:**
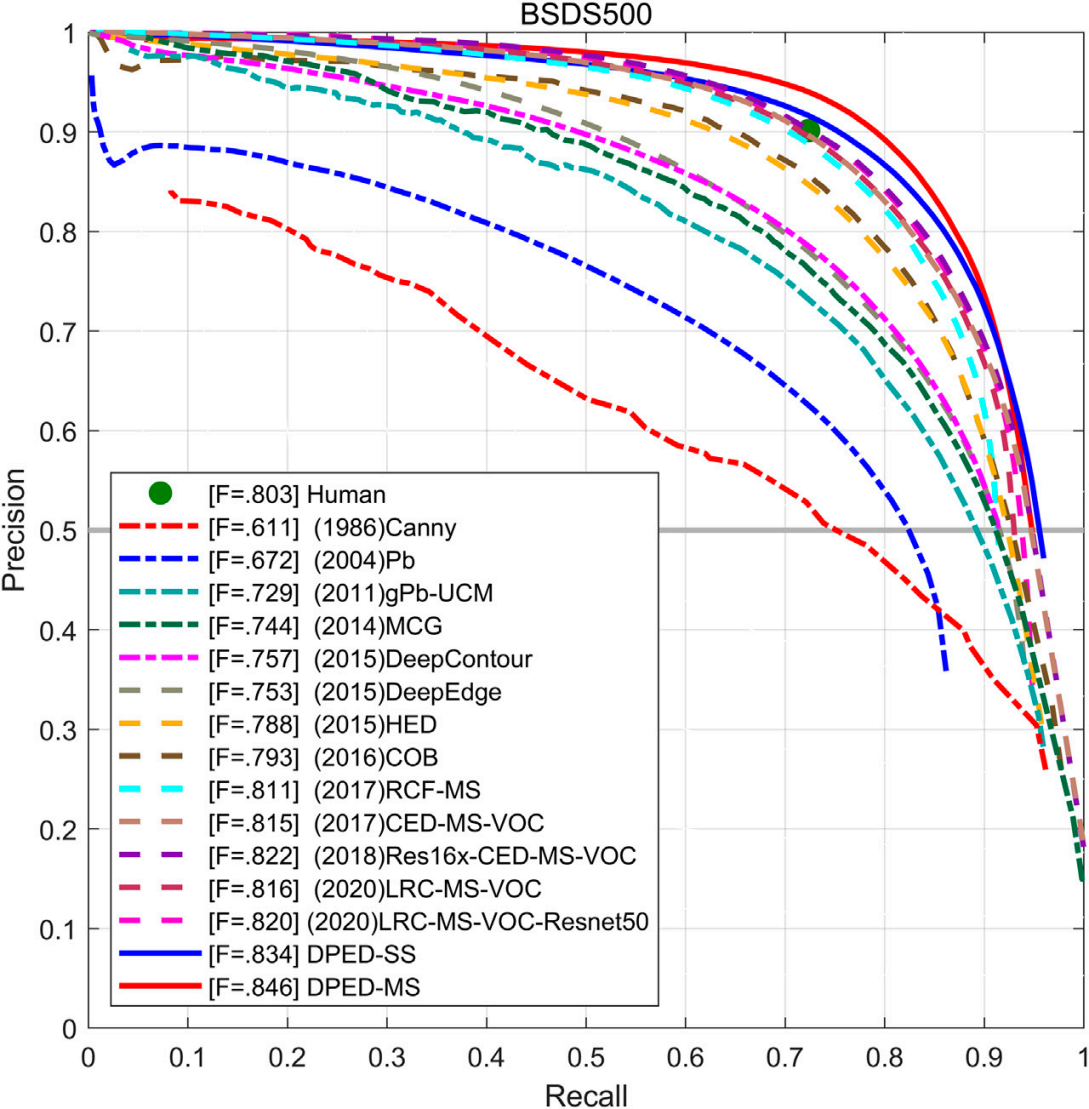
The PR curve of the proposed method and other methods on BSDS500.

The experimental results are summarized in Table 5. The PR curve is shown in Figure 9. The experimental results show that the proposed method achieves the best performance without VOC training. Without additional VOC dataset training, our method achieves single-scale ODS = 0.834 and multi-scale ODS = 0.846, which is higher than the single- and multi-scale ODS of BDCN that is the CNN-based state-of-the-art method. At the same time, the single- and multi-scale ODS of our method is 0.01 and 0.006 higher than the results of EDTER that is the Transformer-based state-of-the-art method. The single-scale OIS and AP of our method also increase in varying degrees. With the addition of VOC dataset training, the single- and multi-scale ODS of our proposed method still exceeds the results of BDCN.

**TABLE 5:** The quantitative results on the BSDS500 dataset. SS represents the test results under single-scale conditions, and MS represents the test results under multi-scale conditions. VOC indicates that the PASCAL VOC context dataset is added to the training set. The first two best effects are marked with red and blue, respectively.

	Method	ODS	OIS	AP
	Human	0.803	0.803	-
**Traditional method**	Canny Canny. (1986)	0.611	0.611	0.611
	SCO Yang et al. (2015)	0.670	0.670	0.670
	SED Akbarinia and Parraga. (2018)	0.710	0.710	0.710
	gPb Arbelaez et al. (2010)	0.729	0.729	0.729
	OEF Hallman and Fowlkes (2015)	0.746	0.746	0.746
	SE Dollár and Zitnick (2014)	0.743	0.743	0.743
**CNN-based method**	COB Maninis et al. (2016)	0.793	0.793	0.793
	HED Xie and Tu (2015)	0.782	0.804	0.833
	RCF-MS-VOC Liu et al. (2017)	0.811	0.830	-
	CED-MS Wang et al. (2018)	0.803	0.820	0.871
	CED-MS-VOC Wang et al. (2018)	0.815	0.833	0.889
	LPCB-MS-VOC Deng et al. (2018)	0.815	0.834	-
	LRC-MS-VOC Lin et al. (2020)	0.816	0.843	0.864
	BDCN-SS He et al. (2020)	0.806	0.826	0.847
	BDCN-SS-VOC He et al. (2020)	0.820	0.838	**0.888**
	BDCN-MS-VOC He et al. (2020)	0.828	0.844	**0.890**
**Transformer-based method**	EDTER-SSPu et al. (2022)	**0.824**	**0.841**	**0.880**
	EDTER-MSPu et al. (2022)	**0.840**	**0.858**	0.896
	EDTER-SS-VOCPu et al. (2022)	**0.832**	**0.847**	0.886
	EDTER-MS-VOCPu et al. (2022)	**0.848**	**0.865**	**0.903**
	DPED-SS(Ours)	**0.834**	**0.850**	**0.884**
	DPED-MS(Ours)	**0.846**	**0.861**	0.838
	DPED-SS-VOC(Ours)	**0.823**	**0.840**	0.832
	DPED-MS-VOC(Ours)	**0.843**	**0.860**	0.775

In our method, the ODS and OIS of multi-scale is much better than that of single-scale, and the multi-scale AP is significantly lower than single-scale AP. We can see from Figure 9 that the single-scale and multi-scale PR curves of our method can cover the PR curve of the latest method. The PR curve of DPED-MS (solid red line) can generally cover the PR curve of DPED-SS (solid blue line) but, at the end of the PR curve, the rapid decline of DPED-MS without enclosing DPED-SS will lead to the decrease of AP. ODS is a more meaningful metric in the field of edge detection. Other research (Cao et al., 2020; He et al., 2020) also considers ODS more important.

To further compare our method with the CNN- and Transformer-based methods, we compared these methods with ODS and frames-per-second (FPS), considering both performance and efficiency. We only compared the public code concrete implementation methods that we could find. The comparison results are summarized in Table 6, where the FPS of EDTER (Pu et al., 2022) are quoted from the original study and calculated on V100 GPU. Limited by equipment conditions, the FPS of other methods is calculated on P100 GPU (worse than V100). It can be seen from the table that the best ODS result of our proposed method is only 0.002 lower than that of EDTER but that our method is more than seven times faster than EDTER on worse GPU.

**TABLE 6:** The comparison of the ODS and FPS of various methods. Among them, the best ODS results obtained by each method are recorded. The FPS is the inference speed on the BSDS500 test set. The FPS of table EDTER are quoted from the original study and calculated on V100 GPU. Due to the limitation of equipment conditions, the FPS data of other methods are calculated on P100 GPU (which is a worse GPU than V100).

Method	ODS	FPS
HED (Xie and Tu 2015)	0.782	93
RCF Liu et al. (2017)	0.811	54
LRC Lin et al. (2020)	0.816	17
BDCN He et al. (2020)	0.828	40
EDTER Pu et al. (2022)	0.848	2.2
DPED (Ours)	0.846	16

**FIGURE 10:**
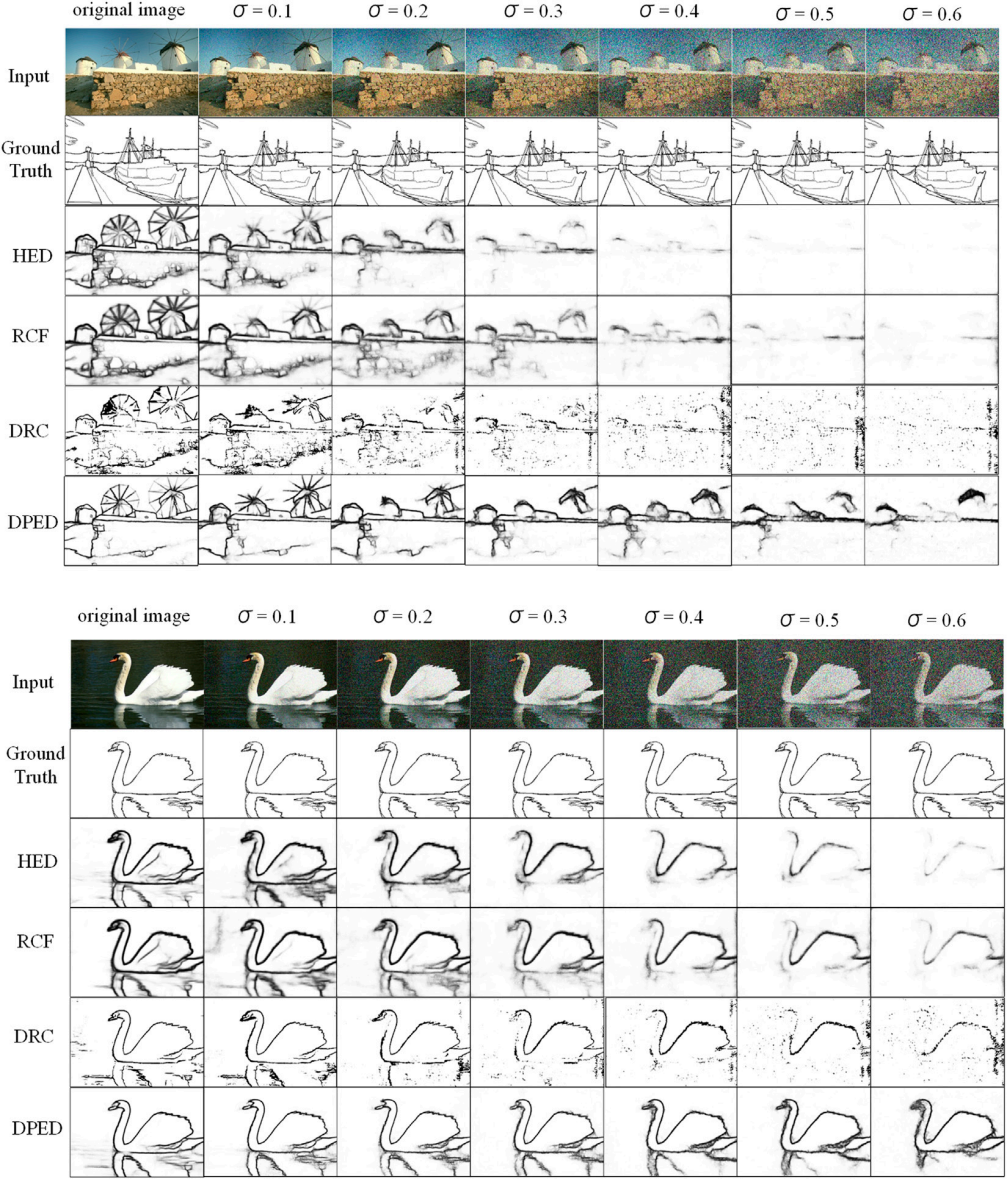
The effect of Gaussian noise with different variances.

The BSDS500 datasets are all high-quality photos, while some traditional algorithms such as Canny can still obtain edge information even when the image quality is reduced. Therefore, we explored the performance of deep learning-based edge algorithms for correctly detecting contours in low-quality images. In the Gaussian function $G\left(x\right)=x+\frac{1}{\sigma \sqrt{2\pi }}e^{\frac{x-\mu }{2\sigma^{2}}}$ , where μ signifies mean and σ means variance, we add different degrees of Gaussian noise to the test set of BSDS500 to create a low-quality test set. We set a zero-mean Gaussian noise test set with variances σ = 0.1, 0.2, 0.3, 0.4, 0.5, 0.6 respectively. We used the peak signal-to-noise ratio (PSNR $=20\log_{10}\left(\frac{1}{\sqrt{\underset{N}{\sum }{\left(x-x^{\prime }\right)}^{2\;}}}\right)$ , where x and $x^{\prime }$ mean the pixels in the original image and the Gaussian-contaminated image. All images are normalized, N signifying the number of picture pixels, to quantify the mean on noise-contaminated datasets and discuss the minimum limit of PSNR for our method. We chose HED, RCF, DRC, and our method DPED for comparative experiments. The ODS metrics of all the methods on different noise datasets are summarized in Table 7 and the visualization of all the methods is shown in Figure 10. Table 7 shows that all methods have performance degradation when adding Gaussian noise to the test, which is caused by the model not seeing samples polluted by Gaussian noise because these methods do not add Gaussian noise in the training. The ODS result of our method is weaker than that of RCF but is better than other methods in the case of Gaussian noise influence with stronger variance. If the ODS result is expected to be no lower than 0.6, our method needs to accept pictures with PSNR ≥ 12.00.

**TABLE 7:** Effects of zero-mean Gaussians with different variances on the performance of deep learning methods. The best ODS results obtained by all methods under different σ are shown in bold. σ=− means the test result without adding Gaussian noise.

Method	σ	-	0.1	0.2	0.3	0.4	0.5	0.6
	PSNR	-	20.38	14.87	12.00	10.27	9.15	8.39
HED Xie and Tu (2015)		0.782	0.716	0.608	0.516	0.414	0.263	0.130
RCF Liu et al. (2017)		0.811	0.739	0.662	0.591	0.520	0.451	0.376
DRC Cao et al. (2020)		0.806	0.650	0.578	0.505	0.421	0.352	0.297
DPED (Ours)		0.834	0.732	0.674	0.612	0.559	0.512	0.471

**FIGURE 11:**
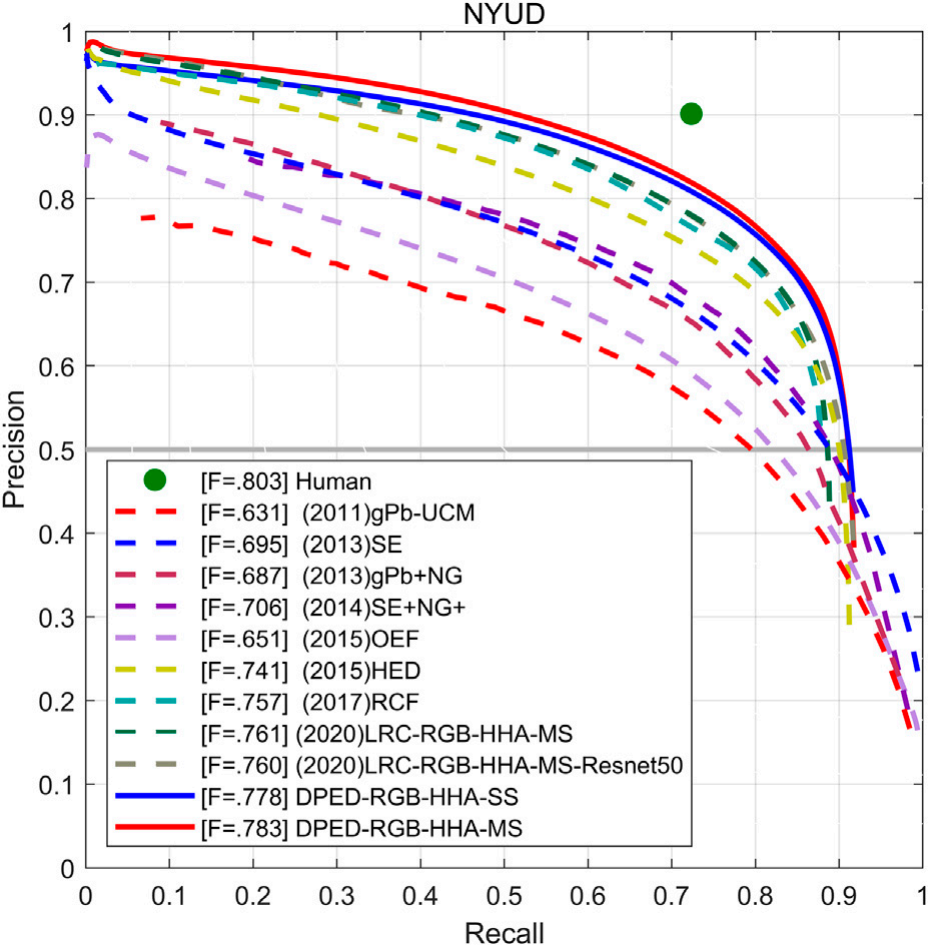
The PR curve of our proposed method and other methods on the NYUD.

**NYUD-v2 Dataset.** We used RGB, HHA, and RGB-HHA to evaluate the network. The results of RGB-HHA are the average of the RGB testing results and HHA testing results. Our method is compared with other methods, including OEF (Hallman and Fowlkes 2015), SE (Dollár and Zitnick 2014), SE + NG+ (Gupta et al., 2014), HED (Xie and Tu 2015), RCF (Liu et al., 2017), LRC (Lin et al., 2020), BDCN (He et al., 2020), and EDTER (Pu et al., 2022). The experimental results are shown in Table 8. Under the three different inputs of RGB, HHA, and RGB-HHA, the single-scale ODS of our proposed method achieves 0.761, 0.709, and 0.778, respectively. The single-scale ODS of our proposed method is 0.013, 0.002, and 0.013 higher than that of BDCN, respectively. Compared with the Transformer-based method EDTER, the HHA ODS result of our method is higher by 0.006, and the RGB and RGB-HHA ODS results of our method are lower by 0.013 and 0.002. The AP of our proposed method is lower than that of BDCN and EDTER. As shown in Figure 11, the PR curve of our proposed method can roughly cover other methods.

**TABLE 8:** The quantization result on the NYUD-v2 dataset. RGB represents the test results of the input RGB images, HHA represents the test results of the input HHA images, and RGB-HHA represents the test results of the average RGB and HHA output. The first two best effects are marked with red and blue, respectively.

	Method	ODS	OIS	AP
**Traditional**	SE Dollár and Zitnick. (2014)	0.695	0.708	0.719
	SE + NG+ Gupta et al. (2014)	0.706	0.734	0.738
	OEF Hallman and Fowlkes. (2015)	0.651	0.667	0.653
	SemiContour Zhang et al. (2016)	0.680	0.700	0.690
**CNN-based**	HED-RGB Xie and Tu. (2015)	0.717	0.732	0.704
	HED-HHA Xie and Tu. (2015)	0.681	0.695	0.674
	HED-RGB-HHA Xie and Tu. (2015)	0.741	0.757	0.749
	RCF-RGB Liu et al. (2017)	0.729	0.742	0.693
	RCF-HHA Liu et al. (2017)	0.705	0.715	0.650
	RCF-RGB-HHA Liu et al. (2017)	0.757	0.771	0.749
	LRC-RGB Lin et al. (2020)	0.743	0.757	0.719
	LRC-HHA Lin et al. (2020)	0.692	0.706	0.668
	LRC-RGB-HHA Lin et al. (2020)	0.761	0.775	0.762
	BDCN-RGB He et al. (2020)	0.748	0.763	0.770
	BDCN-HHA He et al. (2020)	0.707	0.719	**0.731**
	BDCN-RGB-HHA He et al. (2020)	0.765	0.781	**0.813**
**Transformer-based**	EDTER-RGB Pu et al. (2022)	**0.774**	**0.789**	**0.797**
	EDTER-HHA Pu et al. (2022)	**0.703**	**0.718**	0.727
	EDTER-RGB-HHA Pu et al. (2022)	**0.780**	**0.797**	**0.814**
	DPED-RGB (Ours)	**0.761**	**0.774**	0.727
	DPED-HHA (Ours)	**0.709**	**0.722**	0.696
	DPED-RGB-HHA (Ours)	**0.778**	**0.793**	0.791

**Multicue dataset.** Multicue contains two sub-datasets: Multicue boundary and Multicue edge, on which we performed experimental analysis. Our method is compared with other methods, including HED (Xie and Tu 2015), RCF (Liu et al., 2017), DRC (Cao et al., 2020), BDCN (He et al., 2020), and EDTER (Pu et al., 2022). Following this work, we took the results of an average of three independent experiments as the final results. The experimental results are summarized in Table 9. Our method does not perform as well as EDTER on the Multicue boundary dataset but we perform better on the Multicue edge dataset. On the Multicue boundary dataset, the single- and multi-scale ODS results of our method are 0.004 and 0.005 higher than the single- and multi-scale ODS of BDCN, respectively. The single-scale ODS result of our method is 0.021 lower than the single-scale ODS of EDTER. According to the standard deviation of three independent experiments, the ODS of our method is more stable in three independent experiments. On the Multicue edge dataset, our method obtained the single-scale ODS of 0.898 and the multi-scale ODS of 0.900. The single-scale ODS of our method is 0.004 higher than that of EDTER. The single-scale ODS and multi-scale ODS of our method are 0.007 and 0.006 higher than the single-scale ODS and multi-scale ODS of BDCN. In three independent experiments, the stability of the ODS of our method is a little worse than BDCN but a little better than EDTER.

**TABLE 9:** The quantization result on the Multicue dataset. SS represents the test results under single-scale conditions, and MS represents the test results under multi-scale conditions. The data in ( ) represents the standard deviation of three independent experiments. The first two best effects are marked with red and blue, respectively.

Cat.	Method	ODS	OIS	AP
**Boundary**	Human Mély et al. (2016)	0.760 (0.017)	-	-
	Multicue (Mély et al. (2016)	0.720 (0.014)	-	-
	HED Xie and Tu. (2015)	0.814 (0.011)	0.822 (0.008)	0.869 (0.015)
	RCF Liu et al. (2017)	0.817 (0.004)	0.825 (0.005)	-
	DRC-SS Cao et al. (2020)	0.820 (0.006)	0.820 (0.005)	0.710 (0.006)
	DRC-MS Cao et al. (2020)	0.837 (0.001)	0.842 (0.002)	0.786 (0.005)
	BDCN-SS He et al. (2020)	0.836**(0.001)**	0.846**(0.003)**	0.893**(0.001)**
	BDCN-MS He et al. (2020)	0.838 (0.004)	0.853 (0.009)	0.906 (0.005)
	EDTER-SS Pu et al. (2022)	**0.861(0.003)**	**0.870(0.004)**	**0.919(0.003)**
	EDTER-MS Pu et al. (2022)	-	-	-
	DPED-SS(Ours)	**0.840(0.001)**	**0.854(0.004)**	**0.899(0.001)**
	DPED-MS(Ours)	0.843 (0.001)	0.856 (0.004)	0.913 (0.002)
**Edge**	Human Mély et al. (2016)	0.750 (0.024)	-	-
	Multicue Mély et al. (2016)	0.830 (0.002)	-	-
	HED Xie and Tu. (2015)	0.851 (0.014)	0.864 (0.011)	-
	RCF Liu et al. (2017)	0.857 (0.004)	0.862 (0.004)	-
	DRC-SS Cao et al. (2020)	0.859**(0.002)**	0.862**(0.001)**	0.768 (0.010)
	DRC-MS Cao et al. (2020)	0.869 (0.002)	0.873 (0.002)	0.868 (0.002)
	BDCN-SS He et al. (2020)	0.891**(0.001)**	0.898**(0.002)**	0.835 (0.002)
	BDCN-MS He et al. (2020)	0.894 (0.002)	0.901 (0.004)	0.941 (0.005)
	EDTER-SS Pu et al. (2022)	**0.894** (0.005)	**0.900** (0.003)	**0.944(0.002)**
	EDTER-MS Pu et al. (2022)	-	-	-
	DPED-SS (Ours)	**0.898** (0.003)	**0.901** (0.005)	**0.943** (0.006)
	DPED-MS (Ours)	0.900 (0.004)	0.907 (0.004)	0.953 (0.002)

## 5 Conclusion

In this study, inspired by the study of biological vision, we propose a dual-pathway edge detection network—DPED, consisting of a first-pathway network (FPN) and a second-pathway network (SPN). According to research on the attention mechanism in the visual system, we transformed the feature information extracted by SPN into early attention through SPAM, which can work together with late attention (self-attention) in FPN to increase encoding efficiency. We also fused the features extracted by FPN and SPN through the pathways fusion module (PFM) to obtain the pathway fusion feature. To make full use of this feature, we designed a decoding module called FFM to build a decoding network to decode the pathway fusion feature. We conducted experiments on three datasets, including BSDS500 (without VOC), NYUD-v2 and Multicue. Our method surpasses the recent CNN-based method on all datasets. Compared with the recent Transformer-based method EDTER, while some results of our methods have a little lower performance, our method has a lower computational cost and faster inference speed. Our method brings new ideas to edge detection, combining deep learning and biological vision research to design network architectures or modules to improve the performance of edge detection methods.

## Data Availability

The original contributions presented in the study are publicly available. This data can be found here: https://github.com/cimerainbow/DPED.
